# Postextraction Alveolar Ridge Preservation: Biological Basis and Treatments

**DOI:** 10.1155/2012/151030

**Published:** 2012-06-12

**Authors:** Giorgio Pagni, Gaia Pellegrini, William V. Giannobile, Giulio Rasperini

**Affiliations:** ^1^Unit of Periodontology, Department of Biomedical, Surgical and Dental Sciences, University of Milan, Foundation IRCCS Cà Granda, 20142 Milan, Italy; ^2^Department of Periodontics and Oral Medicine and Michigan Center for Oral Health Research, Ann Arbor, MI, USA; ^3^Department of Biomedical Engineering, College of Engineering, University of Michigan, Ann Arbor, MI, USA

## Abstract

Following tooth extraction, the alveolar ridge undergoes an inevitable remodeling process that influences implant therapy of the edentulous area. Socket grafting is a commonly adopted therapy for the preservation of alveolar bone structures in combination or not with immediate implant placement although the biological bases lying behind this treatment modality are not fully understood and often misinterpreted. This review is intended to clarify the literature support to socket grafting in order to provide practitioners with valid tools to make a conscious decision of when and why to recommend this therapy.

## 1. Introduction

Anatomical changes and physiological processes taking over tooth extraction were studied in the past [1–3]; however, since the introduction of dental implants in modern odontology, these issues and the prevention of edentulous jaw atrophy have become very hot topics. The survival of implants and their ability to provide adequate function and esthetic are strictly correlated with their proper positioning in relation to the alveolar housing, the neighboring teeth and the occluding dentition. It is thus easily understood the tremendous effort that has been used by many researchers and practitioners in reducing this unavoidable modeling and remodeling process. This article goes through the biological basis for socket augmentation procedure and the available treatment options to prevent edentulous ridge atrophy.

## 2. Alveolar Ridge Remodeling

Maxillary and mandibular bony complexes are composed by several anatomical structures with a proper function, composition, and physiology: (i) basal bone that develops together with the overall skeleton, and forms the body of mandible and maxilla; (ii) alveolar process that develops following tooth eruption and contains the tooth alveolus; (iii) the bundle bone that lines the alveolar socket, extends coronally forming the crest of the buccal bone, and makes part of the periodontal structure as it encloses the external terminations of periodontal fibres (Sharpey's fibers).

After tooth extraction, bundle bone appears to be the first bone to be absorbed [4–6] whereas alveolar bone is gradually absorbed throughout life [7, 8]. The remodeling process results in a ridge morphology reduced in vertical height and more palatal in relation to the original tooth position [1–3, 9].

**Figure 1:**
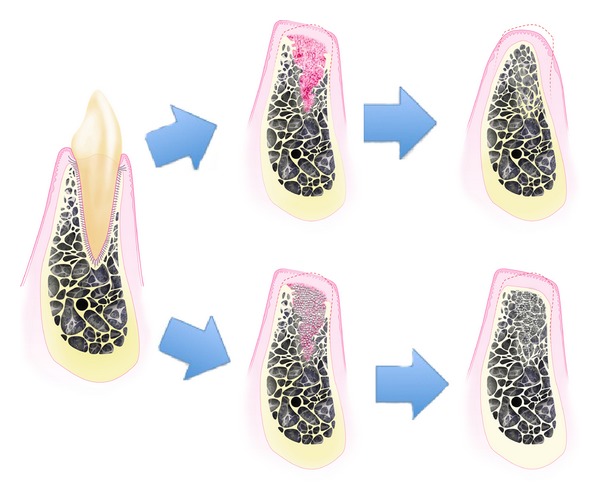
Healing of the extraction socket with and without socket grafting. When socket grafting is not adopted, major alveolar ridge resorption occurs. In a first phase, initially the blood clot, subsequently the granulation tissue and later the provisional matrix and the woven bone fill up the alveolus. The bundle bone is completely resorbed causing a reduction in the vertical ridge. In a second phase, the buccal wall and the woven bone are remodeled causing the horizontal and further vertical ridge reduction. When socket grafting is adopted, the first phase and vertical bone reduction still occur, however, the second phase and the horizontal contraction are reduced.

Studies from another research group suggest bone resorption to occur in 2 phases (see Figure 1). During the first phase, bundle bone is rapidly resorbed and replaced with woven bone leading to a great reduction in bone height especially in the buccal aspect of the socket, as its crestal portion is comprised solely of bundle bone [10]. The buccal plate experiences more resorption even because it is generally thinner, averaging 0.8 mm in anterior teeth and 1.1 mm in premolar sites [11]. *In-vitro* animal studies have demonstrated the osteogenic potential of PDL-derived cells [12, 13] although the role of bundle bone in providing cells for the regeneration of new bone has been more recently challenged [14] as new bone formation appears to initiate from the surrounding alveolar bone cells [4–6]. This group reported that the presence or absence of PDL in the extraction socket does not influence the features of healing after 3 months [15]. During the second phase, the outer surface of the alveolar bone is remodeled causing an overall horizontal and vertical tissue contraction. The reason for this remodeling process is still not well understood. Disuse atrophy, decreased blood supply, and localized inflammation might play important roles in bone resorption. However, it is now apparent that bone remodeling is a complex process involving structural, functional, and physiologic factors and that surgical trauma from extraction induces microtrauma to surrounding bone, which accelerates bone remodeling [16].

Resorption rate of the alveolar ridges is faster during the first six months following the extraction [9, 17] and proceeds at an average of 0.5–1.0% per year for the entire life [7, 8]. The height of a healed socket never reaches the coronal level of bone attached to the extracted tooth, and horizontal resorption seems to be greater in the molar region compared to the premolar area [18, 19]. Schropp et al. estimated two thirds of the hard and soft tissue changes occur in the first 3 months. The authors reported 50% of crestal width to be lost in a 12-month period (corresponding to 6.1 mm; range 2.7 to 12.2 mm), 2/3 of which (3.8 mm; 30%) occurred in the first 12 weeks. When examining the premolar area only, a loss of alveolar ridge width of 4.9 mm (45%) was reported, of which 3.1 mm (28.4%) occurred in the first 12 weeks [20]. A recently published systematic review [21] reported a greater horizontal alveolar ridge reduction (29–63%; 3.79 mm) than vertical bone loss (11–22%; 1.24 mm on the buccal, 0.84 mm on mesial, and 0.80 on distal sites) at 6 months. In a long-term study, Ashman reported an alveolar bone shrinkage of 40–60% in height and width within the first 2-3 years [8, 22].

## 3. Socket Healing

Immediately after tooth extraction, the alveolar socket is filled by blood clot that is replaced by granulation tissue within 1 week (see Figure 1) [23]. In the healing of a skin wound, epithelial cells migrate underneath and are protected by the blood clot. In socket healing instead, the epithelium migrates over the granulation tissue to cover the healing socket [24]. This happens because this inflammatory tissue is recognized as a connective tissue by the epithelial cells, therefore, cellular migration occurs over its surface. This is important when we examine guided bone regeneration applied to socket grafting. Starting from the apical and lateral residual bony walls, the granulation tissue is rapidly remodeled to provisional matrix. Mineralizing processes occur leading to the formation of woven bone that eventually is replaced by mature lamellar bone [25]. For more information on socket healing stages, please refer to Table 1.

Early human histological investigations reported that extraction sockets are filled with delicate cancellous bone in their apical two thirds at 10 weeks, and they are completely filled with bone at 15 weeks [24]. Increased radiopacity is demonstrated as soon as 38 days and radiopacity similar to that of the surrounding bone at 105 days [24]. These figures might be partially biased as specimens were harvested from cadavers; therefore their late age and their systemic condition might have led to delayed wound healing capabilities. On the other side, animal studies demonstrate accelerated healing as 3 weeks old extraction sockets in humans compare with 9-10 days old sockets in dogs and a 3.5 months sockets in humans compares with 8 weeks sockets in dogs [26]. 

## 4. Rationale for Extraction Socket Preservation 

Bone formation in the alveolar socket is a naturally occurring event as long as surrounding alveolar walls remain intact; however, the alveolar ridge volumetric contraction may impair implant placement.

To reduce loss of alveolar bone to acceptable levels, several surgical techniques have been proposed. Reducing the extraction trauma and limiting flap elevation [31] are essential for obtaining success in each of these procedures. Animal studies show mixed results when evaluating differences in ridge remodeling between flapped and nonflapped extraction sockets [31–36] although it has been hypothesized that by disrupting the thin layer of cells that comprises the osteogenic layer of the adult periosteum, the elevation of a flap might diminish the ability of periosteal cells to regenerate bone, while an undisturbed periosteum maintains its osteogenic potential [10, 37–39]. It is possible that flap elevation affects alveolar dimensional alterations only in the short-term [21], while in the long term no appreciable differences are found [36]. In guided bone regeneration 4, methods can be used to increase the rate of bone formation and to augment bone volume: osteoinduction by the use of appropriate growth factors; osteoconduction, where a grafting material serves as a scaffold for new bone growth; distraction osteogenesis, by which a fracture is surgically induced and bone fragments are then slowly pulled apart; finally, guided tissue regeneration, which allows spaces maintained by barrier membranes to be filled with new bone [40]. Utilizing these concepts, it has been proposed guided bone regeneration with nonresorbable and absorbable membranes, several types of bone grafts with or without use of barrier membranes or the addition of mucogingival treatments, and more recently the use of bioactive molecules for the generation of bone in the extraction socket. When analyzing the results of the following described studies, it should be kept in mind the goal of the additional service that is provided to the patient, which include the following:

- to enable installation and stability of a dental implant,
- to reduce loss of alveolar bone volume,
- to reduce need for additional bone grafting procedures,
- to enable the generated tissues to provide implant osseointegration,
- to improve the esthetic outcome of the final prosthesis,
- to regenerate bone faster allowing earlier implantation and restoration.

In the following sections, several articles attempting to obtain these purposes by means of alveolar ridge preservation will be reviewed and briefly summarized.

### 4.1. Ridge Preservation with Membranes

Guided bone regeneration (GBR) techniques utilize barrier membranes to refrain gingival cells from penetrating into the defect to be regenerated. The concept of compartmentalization was introduced by Melcher [39] to explain periodontal wound healing, but it may not be applicable to socket healing. If it were, one would expect the socket to be filled with soft tissue in all instances. On the other side, even early observations in humans and animals demonstrated that the alveolar socket tends to heal by regeneration of bone up to the alveolar crest. As in periodontal wound healing [41–43], the stability of the blood clot previously described explains why the compartmentalization concept does not result in a socket filled by epithelium and how epithelial cells migrate over the granulation tissue to close the healing socket. Questions remain as to whether barrier membranes have an effect in maintaining alveolar ridge morphology.

In 1997, Lekovic and coworkers adopted nonabsorbable ePTFE membranes for the preservation of the alveolar ridge following tooth extraction. No changes in clinical measures were noted in the test sites that remained protected for 6 months while significant volumetric changes were observed in control sites and in test sites experiencing membrane exposure [44]. Pinho and coworkers evaluated the use of a titanium membrane with or without autologous bone graft. They found no significant differences between groups and, therefore, concluded that space maintenance is more important than the use of grafting materials in the treatment of extraction sockets [45].

Barrier membranes seem to minimize alveolar bone resorption when compared to nonintact (released) periosteum regardless of the use of additional grafting material. Titanium membranes certainly would have a distinctly different mechanism of action when compared to resorbable membranes that on the other side reduce the potential of exposure and do not require a second surgical intervention for their removal. In 1998, Lekovic et al. examined the effect of glycolide and lactide polymer membranes demonstrating reduced loss of alveolar height, more internal bone socket bone fill and less horizontal resorption than controls [46]. Luczyszyn et al. evaluated the effect of acellular dermal matrix with or without a resorbable hydroxylapatite graft. Both groups preserved ridge thickness, although, better results were achieved in the combined treatment group suggesting that bone grafts might benefit bone regeneration when using a resorbable membranes [47].

A recent study performed a detailed evaluation of the healing of extraction sockets covered with a resorbable collagen membrane. Through the use of histological evaluation, subtraction radiography, and of *μ*-CT analysis, this study demonstrated that adequate bone formation for implant placement occurs as early as 12 weeks following tooth extraction, with insignificant changes in alveolar ridge dimensions [48].

### 4.2. Ridge Preservation with Bone Grafts and Bone Substitutes

The clinical advantages of bone fillers in alveolar ridge volume preservation and prevention of additional bone grafting procedure are largely supported by the available literature [47, 49–51]. Minimal ridge remodeling has been observed when using nonresorbable hydroxyapatite crystals covered by a rotated pediculated split thickness palatal flap [52], DFDBA covered with an ePTFE membrane [53], or even allogenic or xenogenic bone grafts covered with nothing but a collagen plug [51, 54] (Figure 1). Histological evidence demonstrates that bone formation occurs over the surface of the implanted osteoconductive graft particles [55, 56]. At 3 months or later, grafted sockets generally demonstrate higher mineralized tissue figures, when considering both new vital bone and remaining graft particles, but the formation of new bone appears to be similar in grafted and nongrafted sites. It can be extrapolated that residual particles occupy part of the volume that would have been occupied by bone marrow if bone grafting were not adopted [57].

At earlier healing stages (2 weeks) instead, grafted sockets demonstrate xenograft particles enclosed in connective tissue and coated by multinucleated cells when nongrafted sites already show newly formed woven bone occupying most of the socket [58]. This response is typical of a foreign body reaction which can be elicited by the xenograft and though it is clinically non-immunogenic, non-toxic and chemically inert [59], it results in a delayed healing response during the earliest stages of socket healing. Many articles reported only a partial resorption of the grafted particles at short and long timepoints [49, 53, 58, 60–63] arising doubts on the achievement of the osteointegration of implants inserted in augmented sites and on the success of the restorative therapy. Histological animal studies [64, 65] evaluated the osteointegration of dental implants following bone regeneration performed with different bone fillers and observed a bone-to-implant contact similar to that of implants placed in pristine bone (40% to 65%). Furthermore, clinical studies observed that good primary stability can be reached at implant insertion, that the grafting procedure does not impair early osteointegration [66, 67], and that implants placed in bone regenerated using mineralized grafts are able to sustain loading and provide similar long-term results as those placed in pristine bone [68].

Mineralized grafting materials may interfere with the earliest stages of socket healing and their elimination may require several years [57] or they may in fact be nonresorbable even in the long term [62]. On the other side, their ability to prevent crestal ridge resorption and sustain long-term implant success has been clearly demonstrated [66–68].

Other advantages in the use of osteoconductive grafting material were reported by a clinical and histological human study of postextractive defects in posterior maxillary area treated with a xenogenic graft. In this study, Rasperini et al. confirmed the space-maintaining activity of the implanted material and reported a decreased demand for sinus lift augmentation procedure when the socket preservation procedure was performed [63]. Through a computed tomography analysis of maxillary anterior postextractive defects, Nevins et al. reported that 79% of grafted sites underwent less than 20% buccal plate loss, while 71% of nongrafted sites demonstrated more than 20% buccal plate loss. An interesting finding of this investigation was that even the experienced surgeons participating to this study were not able to predict the fate of the buccal plate, therefore, the authors suggested socket grafting to be performed at the time of extraction [69]. 

#### 4.2.1. Buccal Bone Overbuilding

Another technique that may be adopted is to augment the buccal bone by implanting graft materials on its buccal surface. Simon et al. used DFDBA covered by a bioabsorbable membrane for the augmentation procedure. The dimensions on the ridge were augmented compared to the original volume but the invasiveness and technical demand of this procedure may refrain the clinician from its use in everyday practice [70]. In another study, 2 different grafting techniques were adopted according to whether the buccal bone was intact or dehiscence. Sockets with an intact buccal bone were grafted to the level of the alveolar crest, a membrane was used to protect the defect, and the flap was closed by primary intention while sockets with deficient buccal bone were augmented. Their results showed complete loss of the horizontally augmented bone in augmented sites, but grafted sited experienced bone loss in a greater extent than augmented sites [71].

An histological animal study found that buccal bone augmentation with a xenograft failed to prevent the physiological bone modeling and remodeling taking part in the buccal and lingual bony walls; however, the insertion of grafting material seemed to promote *de novo* hard tissue formation, thus limiting the total bone volume contraction [57]. Xenograft particles positioned on the buccal surface of the extraction alveolus were found to be encapsulated in collagen fibers after 3 months of healing. They were always located lateral to the periosteum of the buccal wall and, therefore, did not participate to ridge augmentation [57]. Fickl and coworkers also proposed the overbuilding of the buccal bone with a xenograft and a membrane. Data from their studies indicates that extrasocket grafting does not seem to compensate for ridge alteration after extraction possibly because of the additional trauma to buccal tissues [72, 73].

#### 4.2.2. Free Soft-Tissue Grafts over Grafted Sockets

The placement of free soft-tissue graft to cover the augmented alveolar socket was introduced to minimize the soft tissue shrinkage, optimize aesthetical results of implant restoration, and obtain a primary closure that may preserve the graft from bacterial infections and secondary graft failure [74, 75]. The first attempt to cover the socket graft with an autogenous soft tissue implant was described by Landsberg and Bichacho in 1994 [76]. Nevins and Mellonig suggested the use of soft tissue grafts to improve ridge topography after tooth extraction [77] and in combination with immediate implant placement [78].

In 1999, Tal described the survival of circular connective tissue grafts placed over extraction sockets treated either with DFDBA or Bio-Oss. They found that the survival was not dependent on the adopted graft and that at 1 week 18/42 grafts were vital, 13/42 were partially vital, and 11/42 were nonvital. Complete closure of all sockets occurred 4 weeks postextraction. The authors noted that more often partially vital grafts maintained their vitality over the socket area more than on the graft margins; they concluded that the nourishment could be originated from plasmatic elements in the socket blood clot more than from vessels originating from the periphery of the graft [79].

### 4.3. Immediate Implant Placement and the “Jumping Distance.”

The first report of implant placement immediately after tooth extraction dates back to 1978 when the Tübingen immediate implant was described [80–82]. In 1991, Barzilay et al. suggested that immediate implant placement might reduce or eliminate alveolar ridge resorption during the initial healing of the alveolar extraction socket [83]. In two subsequent papers in a monkey model, he demonstrated that substantially less ridge remodeling was induced in the immediate implant group [84] and that histologically bone to implant contact was similar within the different anatomic regions of the oral cavity [85].

**Figure 2:**
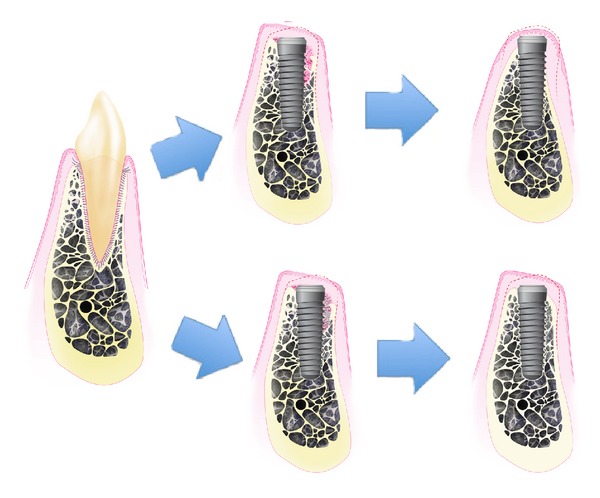
Healing of the extraction socket, with postextractive implant placement, with and without socket grafting. After tooth extraction and immediate implant placement, the blood clot fills the remaining space and the bundle bone undergoes the physiological changes. When grafting material is placed around the implant surface, filling the remaining socket area, the buccal bone wall remodeling process is corrupted, thus leading the maintenance of the horizontal ridge volume.

Other authors challenged the results of the Canadian reporting that the placement of an implant in the fresh extraction site failed to prevent the remodeling that occurred in the walls of the socket. The height of the buccal and lingual walls at 3 months was similar compared to extraction only sites [86–90]. Vertical bone loss was more pronounced at the buccal aspect even with some marginal loss of osseointegration [87]. Histologically, the gap between the implant and the socket walls filled in at 4 weeks with woven bone, while, the buccal and lingual walls underwent marked surface resorption. After 12 weeks, the buccal crest was located >2 mm apical of the implant margin [88] (Figure 2). Evaluating immediately placed implants, Schropp et al. reported 70% of the 3-wall infrabony defects with a parallel width of up to 5 mm, a depth of maximum 4 mm, and a perpendicular width of maximum 2 mm had a capacity of spontaneous healing within a period of 3 months [18]. Botticelli et al. found that 1–1.25 mm wide and 5 mm deep defects around implants healed uneventfully with or without membrane [91]. Defects up to 2.25 mm wide were found to heal using barrier membranes, although when the buccal bone was intentionally removed, less regeneration at the buccal aspects was observed [92]. These studies adopted an animal model with surgically created defects, which typically exhibit lesser resorption than extraction sockets [90].

When immediate implant placement is adopted, many clinicians feel the need of “filling” the buccal gap (i) by placing a larger diameter implant, (ii) by placing the implant in a more buccal position, or (iii) by grafting the buccal defect with some kind of bone substitutes. Given the available literature, the first two strategies do not seem to be recommendable. It seems instead that the presence of a large gap between the buccal wall and the implant apparently promotes new bone formation and enhances the level of bone-to-implant contact [88].

An implant position 0.8 mm deeper and more lingual in relation to the center of the socket results in a lesser degree of buccal bone dehiscence [93]. Other studies demonstrated that the closer the implant is to the buccal bony plate, the more the buccal bone resorbs [94, 95]. Bone resorption of the buccal crest is more pronounced when placing large size (5 mm) root-formed implants when compared to cylindrical implants with a smaller diameter (3.3 mm) demonstrating that implants placed immediately after tooth extraction fail to preserve the alveolar crest of the socket irrespective of their design or configuration [96]. Moreover, soft tissues followed bone levels and also they were located more apical on large size implants compared to smaller size implants [97].

Caneva et al. evaluated the use of a collagen membrane over the buccal gap of immediately placed implants and found that the alveolar crest outline was better maintained at the test sites compared with the control sites even if the buccal gap was relatively small [98]. Interestingly, enhanced bone preservation was found when using deproteinized bovine bone mineral particles and a collagen membrane compared to controls whereas no such benefit was noted when using magnesium-enriched hydroxyapatite [99–101]. Recently Araújo and coworkers have evaluated the use of Bio-Oss Collagen in the volume between the buccal wall and the implant in cases treated with immediate implant placement in an experimental animal model. The authors found that this treatment modified the process of hard tissue healing, provided additional amounts of hard tissue at the entrance of the previous socket, improved the level of marginal bone-to-implant contact, and prevented soft tissue recession [102] (Figure 2).

Implants immediately placed into fresh extraction sockets are classified as Type 1 implants, early placed implants (4–8 weeks) following tooth extraction are Type 2 implants, Type 3 implants represent implants early placed (12–16 weeks) in a socket with partial bone healing, and Type 4 implants are delayed implants placed in a fully healed edentulous site (>6 month) [103]. Timing of implant placement is not a topic to be treated in this review but it might be of interest to the reader that bone grafting in early placed implants (Type 2-3) seems to provide better hard tissue dimensions and with less postoperative complications than bone grafting in delayed implants (Type 4) [104].

When evaluating the expression of osteogenesis-related growth factors, Lin et al. demonstrated apparent tissue maturation delayed during osseointegration, compared to extraction socket bone repair. The two healing models developed distinct features and triggered a characteristic coordinated expression and orchestration of transcription factors, growth factors, extracellular matrix molecules, and chemokines. These groundbreaking findings open new horizons to researchers, which might lead to a better understanding of the cooperative molecular dynamics in alveolar bone healing [105].

### 4.4. Ridge Preservation with Nonmineralized Grafts

Serino et al. evaluated the use of a bioabsorbable polylactide-polyglycolide acid sponge as a ridge preservation grafting material. The grafting material was placed with no attempt to achieve primary intention wound closure. 6 months following the extractions, biopsies were harvested. Both test and control extraction sockets showed mature and well-structured bone with no residual particles of the grafted material. Clinical measures seemed to favor the test group [106]. In a following study, both the regenerated sites and controls resulted in the formation of a highly mineralized and well-structured bone with the control group showing a “slightly minor percentage of mineralized bone” and a higher presence of connective tissue in the coronal portion of the biopsies. Particles of the grafted material could not be identified in any of the biopsies [107].

Grafting materials with high resorption rates allow for the formation of bone with no residual graft particles at the time of implant placement and loading but their ability to sustain alveolar ridge volume in the long term might be inferior to that of mineralized grafts.

### 4.5. Novel Tissue Engineering Approaches

In order to overcome the limitations of routinely adopted biomaterials as allografts, xenografts, and alloplasts in terms of predictability and quality of bone formation and ability to sustain alveolar ridge morphology over long periods of time, novel tissue engineering therapies have been developed including the delivery of growth factors incorporated in carriers, the stimulation of the selective production of growth factors using gene therapy, and the delivery of expanded cellular constructs.

Bone morphogenic proteins (BMPs) are an example of growth factors; they have the ability of inducing the differentiation of the host stem cells into bone forming cells in a process known as *osteoinduction *[108]. A feasibility study introducing the use of rhBMP-2 absorbed in a collagen sponge for alveolar ridge preservation after tooth extraction was published in 1997. Howell et al. demonstrated the safety of this grafting material. Patients receiving socket grafting demonstrated increase in bone height while patients receiving a ridge augmentation procedure showed no evidence of augmented ridge width or height [109]. Implants placed in the regenerated bone were stable and presented healthy periimplant tissues [110]. After this pilot study, Fiorelini and coworkers performed a randomized clinical trial testing the regenerative potential of the recombinant BMP-2 in the collagen sponge compared to the use of the collagen sponge alone. Anterior maxillary postextraction alveolar defects in which more than 50% of the alveolar buccal bone had been lost prior to extraction were treated with either of the two grafting material. Two different rhBMP-2 concentrations were used (0.75 mg/mL and 1.50 mg/mL). Significantly greater augmentation was noted in the 1.50 mg/mL group and both rhBMP-2 groups outperformed the control groups. Histological findings showed generation of bone no different from native bone [111].

PDGF-BB in a *β*-TCP carrier is a material accepted from the FDA for regeneration of bone and PDL elements in guided tissue regeneration procedures. Nevins et al. evaluated the use of the recombinant protein in socket grafting. In this case, series 8 extraction sockets received Bio-Oss Collagen hydrated with 0.3 mg/mL PDGF-BB, and flaps were released for closure by primary intention. Then 4 or 6 months after grafting bone core, biopsies revealed “robust bone formation”. Also 23.2 ± 3.2% new bone and 9.5 ± 9.1 residual grafting material were noted at 4 months. However, 18.2 ± 2.1% new bone and 17.1 ± 7.0% residual grafting material were noted at 6 months in the hystomorphometrical evaluation [112]. More recently, tissue repair cells (TRC), a cell construct derived from each patient's bone marrow and cultivated using automated bioreactors to concentrations not achievable through a simple bone marrow aspiration, were evaluated in socket healing. This study showed that this cell construct is able to produce significant concentrations of cytokines and maintains the cells' ability to differentiate toward both the mesenchymal and endothelial pathway and produce angiogenic factors. TRC therapy enhanced formation of highly vascular mature bone as early as 6 weeks after implantation when compared to guided bone regeneration with no serious study-related adverse event reported and lower degrees of alveolar ridge resorption were noted [113, 114]. Please refer to our recent review for further information on cell therapy applications in craniofacial regeneration [115].

## 5. Conclusions

Postextraction alveolar ridge resorption is an inevitable process and the molar area is not an exception. Molar ridges present higher degrees of resorption than premolar areas do. Socket grafting techniques have been readily adopted by dentists throughout the world. A great amount of research has been conducted to examine the effectiveness of several materials or membranes.

The use of invasive techniques is hardly recommended at this treatment timepoint as any procedure requiring primary intention healing with the advancement of flaps may result in increased inflammatory response, in a decrease in vestibular depth, and in the creation of unaesthetic scars. Even expert practitioners might not be able to accurately determine when these techniques might be indicated [69]. For the very same reason, less invasive grafting techniques should be adopted when indicated especially when treating defects in the esthetic or molar areas. It should be understood that the use of osteoconductive-mineralized grafts does not accelerate bone healing, but may allow for a better preservation of the ridge volume that is highly desirable for both esthetic and function of the future implant restoration. Moreover, invasive procedures as guided bone regeneration and sinus floor elevation are less frequently needed when socket grafting is adopted [63]. For more predictable results, it is recommended to allow proper time for bone healing prior to proceed with implant placement. Anyway, when immediate implant placement is adopted, the use of mineralized grafts on the buccal gap helps reducing the resorption of the buccal crest bone [102] and may lessen the chances for undesirable hard and soft tissue recessions. Clinicians should escape the temptation of placing larger diameter implants or placing the implant in a more buccal position in order to fill the buccal gap. Instead, a larger gap should be preserved and the buccal defect should be filled with bone substitutes.

The rationale for a frankly palatal/lingual positioning of immediately placed implants is also supported by the knowledge that significantly more facial recessions are correlated with implants placed too buccal [116, 117].

Advances in tissue engineering techniques might soon provide practitioners with biomaterials for a more predictable and enhanced bone formation that will definitely improve our clinical results. These novel biomaterials are currently evaluated worldwide and will soon be introduced in everyday practice.

Practitioners should be well informed of the biological characteristics of new biomaterials and on which stages of wound healing may they take an action.

This paper attempted to summarize the concepts on socket grafting resulting from the available literature. Current knowledge may still be insufficient to fully justify the use of certain techniques in everyday practice, and more studies evaluating basic biological concepts should be performed.

In socket grafting as in other medical divisions, proper diagnosis is often more important than the rendered treatment.

## Figures and Tables

**Table 1:** Healing of the extraction socket. Articles reporting timing and histological evidence of extraction socket healing events are reported.

Reference	Model	Healing
Clafin, 1936 [26]	Experimental extraction in a dog model.	Day 1. Blood clot filled the socket, fibrin network covered the clot. Day 3. Epithelium starts to proliferate. Osteoclasts are present on the bone crest. Fibroblasts started invading the clot. Day 5. Bone formation at the fundus of the socket. Day 11. New bone along the alveolar socket walls. Day 19. New bone reached the crest. The clot is present in the center of the socket. Day 28. The alveolus is filled with new bone.

Weinmann and Sicher, 1955 [27]	Animal model.	Blood clot. Organization of the blood clot by proliferating connective tissue. Replacement of the connective tissue with fibrillar bone. Reconstruction of the coarse fibrillar bone and replacement by mature bone matrix.

Amler et al., 1960 [28]	Human biopsies of the content of extraction wounds scooped out with small curets. 3 days intervals.	Clot formation. Replacement with granulation tissue (7th day). Replacement of granulation tissue with connective tissue (20th day). Osteoid is present at the base of the socket at the 7th day and fills 2/3 of the socket at the 28th day. Epithelialization starts on the 4th day and is complete after day 24. Epithelial migration proceeds from the margins of the socket with the organization of the clot.

Boyne, 1966 [4]	12 patients (20–45 yo). Extraction of 1st maxillary premolar. Flaps were not elevated. 2 doses of IM Oxytetracyclines at different postoperative days for each patient. 1 week after administration of the antibiotic all the remaining maxillary teeth were extracted and a block section of the whole socket of the 1st premolar is harvested and grafted with FDBA.	Specimens tagged at day 5-6. No fluorescent new matrix. Day 7-8. Fluorescent new bone in the marrow vascular spaces adjacent to and along the entire length of the lamina dura. No bone in the socket. Day 9-10. New bone appears also on the lateral aspects of the socket walls. Day 12. New bone along the lateral walls and in adjacent bone areas. Day 13-14. New bone fills approximately 1/3 of the alveolus. Day 15-16. Similar to previous 13-14 days specimens. Day 19. Bone matrix had filled a large portion of the socket.

Evian et al., 1982 [29]	10 patients. Extractions at different timepoints prior to periodontal surgery. Bone cores harvested at the time of periodontal surgery. Conclusions: 8–12 weeks is the best timeframe in which to harvest a graft.	4 weeks: Abundance of fibrous connective tissue. Rows of osteoblasts in the osteoid layer. 6 weeks: Osteoblasts are actively laying down new bone. 8 weeks: Trabeculae of new bone occupy the majority of the socket. Fewer osteoblasts and less osteoid are present. 10 weeks: Trabeculae interconnected with a minimum of osteoid. 12 weeks: Similar to 10 weeks. 16 weeks: Dense bone trabeculae with fewer cellular elements. Very little bone formation and few osteoblasts.

Hsieh et al., 1994 [5]	Rat teeth are extracted and fluorochrome is administered at different intervals. Conclusions: mineral formation is greatest at the gingivopalatal aspect and least at the gingivobuccal.	5 days: osteogenesis mainly in the apical region. Subperiosteal bone formation on the external surface of the buccal bone. 10 days: epithelium covered the socket. Woven bone filled 1/3 to 1/2 of the socket height. Margins of the socket are rounded by resorption of buccal and palatal crests and the apposition of buccal subperiosteal woven bone. Day 14: thick trabeculae fill the socket. Numerous osteoblasts and few osteoclasts.

Devlin and Sloan, 2002 [6]	Extraction socket of patients requiring mandibular squamous cell carcinoma resection. Extractions were performed 2 weeks prior to resection.	2 weeks postextraction the PDL ligament was present in the center of the socket. Osteocytes and osteoblasts in the marrow spaces and on the socket margins strongly expressed Runx2, pre-osteoblasts on the socket surfaces, osteoprogenitor cells in the center of the socket also expressed Runx2. SB-10 and SB-20 antibodies were expressed in osteoprogenitor cells, pre-osteoblasts and osteoblasts surrounding trabeculae.

Cardaropoli et al., 2003 [30]	9 mongrel dogs (1 for each timepoint). Distal roots of the 4th mandibular premolars are extracted. *B* and *L* soft tissue is stabilized by sutures. Sections in the *M*-*D* direction.	Day 1: Coagulum fills most of the socket, inflammatory cells in the connective tissue. Day 3: Small areas of the coagulum are replaced by richly vascularized granulation tissue. Day 7: The clot is partially replaced by a provisional matrix. Day 14: The socket margins are covered by connective tissue. The socket contained a provisional matrix and woven bone. PDL and bundle bone are absent. Woven bone extends from the socket walls to the center of the wound. Day 30: Osteoclasts are resorbing woven bone and are also observed on the surface of the old lamellar bone of the crestal region. Soft tissue is organized and keratinized. Day 60 and 90: A woven bone hard tissue bridges the defect. Woven bone is being replaced by lamellar bone. Day 120 and 180: Bridging bone is remodeled to lamellar bone. A new periosteum is established.
